# Miscommunication of science: music cognition research in the popular press

**DOI:** 10.3389/fpsyg.2015.00988

**Published:** 2015-07-20

**Authors:** Samuel A. Mehr

**Affiliations:** ^1^Graduate School of Education, Harvard UniversityCambridge, MA, USA; ^2^Department of Psychology, Harvard UniversityCambridge, MA, USA

**Keywords:** science communication, music, public opinion, methods, writing

In December 2013, my colleagues and I published the null results of two randomized trials investigating effects of brief parent-child music enrichment on preschoolers' cognitive skills (Mehr et al., 2013). Fully aware of the limitations of our studies, including, of course, that a failure to reject the null hypothesis does not imply evidence in support of the null hypothesis, we conservatively titled the paper “Two randomized trials reveal no consistent evidence for nonmusical cognitive benefits of brief preschool music enrichment.” In the discussion we wrote over 1000 words on why we might not have detected a positive effect, should one exist (pp. 9–10). Nonetheless, a media firestorm ensued, in which press reports claimed not only that our findings affirmed the null hypothesis, but also that they implied a broader conclusion: music lessons confer no cognitive benefits whatsoever (e.g., regardless of child age or training content, duration, or intensity). For instance, the *Times of London* reported, “Academic benefits of music ‘a myth’” (Devlin, 2013); a *Boston Globe* headline read, “Music doesn't make you smarter, Harvard study finds” (Johnson, 2013); and *TIME* reported “Music can soothe the soul and speed along creativity, but it won't, according to researchers from Harvard, boost intelligence” (Sifferlin, 2013). These headlines appeared alongside 100 other reports from over 40 countries (partial listing: https://goo.gl/pCwvqG), despite efforts to clarify our findings via numerous interviews, a live Q&A on Reddit, and a New York Times op-ed (Mehr, 2013).

Perhaps we should not have been surprised. The idea that “music makes you smarter” is widely accepted by the general public (e.g., Mehr, 2014) and traces back to a sensationalist media interpretation of a *Nature* paper describing improved spatial task performance after listening to a Mozart sonata (Rauscher et al., 1993). This “Mozart effect” has been called a “scientific legend” (Bangerter and Heath, 2004) and was conclusively debunked (e.g., Chabris, 1999; Thompson et al., 2001), but not before it elicited a media frenzy that affected political policy: it was cited in a US House of Representatives debate on public funding of arts programs (Trescott, 1997) and by President Bill Clinton in a speech on arts education (Hershenson, 2000), and even prompted the governor of Georgia to sign a bill funding the distribution of classical music CDs in maternity wards (Bangerter and Heath, 2004).

This sort of sensationalism also occurs in coverage of other scientific fields. A year before our *PLoS* findings were published, Barron and Brown (2012) described in *Nature* misleading media coverage of research on animal sexual behavior. For instance, a study of the evolution of cooperative breeding (Young et al., 2008) was described in the press using language of human sexuality (“The love that daren't squawk its name: when animals come out of the closet”; Mooallem, 2010) despite the study's actual topic of mate choice in the Laysan albatross. Taking Barron and Brown's lead, I reviewed media coverage of recent scientific research in music cognition. I searched through roughly 15 years' worth of articles from the world's fifty highest-circulation print and online publications (Pew Research Center, 2013). Here, I present some descriptive results of my review. A full empirical study of trends in science journalism is outside the scope of a brief commentary, but I hope that this article might prompt such research and also help to stop exaggerated media claims about music cognition research.

I found reporting errors in two categories. First, in many papers of record, journalists fell prey to the *post hoc ergo propter hoc* fallacy, misinterpreting the findings of correlational studies as causal effects. For instance, Hanna-Pladdy and MacKay (2011) compared elderly musicians to elderly non-musicians and found the musicians had stronger cognitive skills. The *Philadelphia Inquirer* reported that musical training was the cause: “Those clarinet lessons helped you tune up for your later years: Getting to Carnegie hall isn't the only reason to practice, practice, practice” (Bauers, 2011). However, the study's retrospective design precluded any causal inference, which the authors stated clearly: “…the correlational design of our studies does not allow us to comment on whether musical participation causally enhanced cognition or whether other variables were responsible for the findings” (p. 384). This measured interpretation fell on deaf ears, as other widely-read publications joined the *Inquirer* in a failure to distinguish correlation from causation: *FOX News, The Huffington Post*, and *The New York Times* reported the result in causal language (FOX News, 2011; Huffington Post, 2011; Klass, 2012).

Second, press reports made critical errors in interpreting psychometric outcome measures. For instance, improvement in word recall after music lessons (Ho et al., 2003) was reported in *The New York Times* under the headline “More music yields more words,” stating, “If you want to improve the vocabulary of your children, sign them up for the orchestra…” (O'Neil, 2003). This is dramatically inaccurate: a word recall test assesses memory, not vocabulary. Similarly, when Fujioka and colleagues reported (Fujioka et al., 2006) that musical training affected children's neural response to sound, the *Daily Mail* reported “Music lessons are an IQ booster for young minds” (Hope, 2006). That study made no mention of IQ. And our *PLoS* findings were reported with “…scientists are not so sure that [music] boosts IQ” on *National Public Radio* (Neuman, 2013), despite our explicit decision not to use IQ as an outcome measure (a decision to which we devoted 300 words of explanation; Mehr et al., 2013, p. 3).

That dry scientific titles are translated into catchy headlines is not necessarily worrisome; after all, science journalism can only thrive if the general public actually reads its journalistic product. However, these catchy headlines often include both error types I have described. For instance, “Voxel-based morphometry reveals increased gray matter density in Broca's area in male symphony orchestra musicians” (Sluming et al., 2002) became “Music improves brain power in some performers,” with the subhead “Mozart increases mental mass” (Radford, 2003). The causal direction was unknown, and gray matter density is not “brain power.” Our *PLoS* paper's title became “Do, Re, Mi, Fa-get the piano lessons: Music may not make you smarter” (Sifferlin, 2013). We studied neither piano lessons nor general intelligence.

I have not attempted to estimate the overall frequency of media misrepresentation of music cognition research, nor have I attempted a systematic comparison of that frequency to that of other fields, and so I cannot comment on the prevalence or relative severity of the problems I describe above. But granting that misrepresentation occurs with nonzero frequency, and that it can occur in such prominent news outlets as those cited above, these descriptive findings raise a sticky question: Whose fault is all this? Are journalists sensationalizing research findings to garner page-views and sell papers, or are scientists exaggerating the importance of their own work?

I speculate that the answer is “both,” and hope that this commentary might encourage music cognition scholars to do what we can to anticipate and avoid such issues when publishing new work. We should not take for granted public understanding of basic principles of scientific inference (e.g., correlation is not causation; effects reported are specific to the intervention that was tested) and in interviews and/or public discussion of our research, we should distinguish clearly between data-driven conclusions and idea-driven speculation. If and when our work is misrepresented, we must engage directly with journalists and with the public to correct the record, rather than throwing up our hands in frustration and keeping quiet. Music cognition research is in a particularly sensitive position when it comes to the press: our studies often involve three topics subject to intense public interest—children, brains, and music—and so we must make every effort to promote accurate and responsible public dissemination of our work.

## Conflict of interest statement

The author declares that the research was conducted in the absence of any commercial or financial relationships that could be construed as a potential conflict of interest.

## References

[B1] Bangerter A. Heath C. (2004). The mozart effect: tracking the evolution of a scientific legend. Br. J. Soc. Psychol. 43, 605–623. 10.1348/0144666042565353 15601511

[B2] Barron A. B. Brown M. J. F. (2012). Science journalism: let's talk about sex. Nature 488, 151–152. 10.1038/488151a 22874947

[B3] Bauers S. (2011, April 25). Those clarinet lessons helped you tune up for your later years. Philadelphia Inquirer, C02.

[B4] Chabris C. (1999). Prelude or requiem for the ‘Mozart effect’? Nature 400, 826–827. 10.1038/23608 10476958

[B5] Devlin H. (2013, December 12). Academic benefits of music ‘a myth.’ The Times of London. Available online at: http://www.thetimes.co.uk/tto/science/article3946476.ece (Accessed 3 July, 2015).

[B6] FOX News (2011, April 22). Childhood music lessons keep aging brain in tune. FOX News. Available online at: http://www.foxnews.com/health/2011/04/22/childhood-music-lessons-aging-brain-tune (Accessed 3 July, 2015).

[B7] Fujioka T. Ross B. Kakigi R. Pantev C. Trainor L. J. (2006). One year of musical training affects development of auditory cortical-evoked fields in young children. Brain 129, 2593–2608. 10.1093/brain/awl247 16959812

[B8] Hanna-Pladdy B. MacKay A. (2011). The relation between instrumental musical activity and cognitive aging. Neuropsychology 25, 378–386. 10.1037/a0021895 21463047 PMC4354683

[B9] Hershenson R. (2000, August 6). Debating the Mozart theory. The New York Times, 22.

[B10] Ho Y. Cheung M. Chan A. S. (2003). Music training improves verbal but not visual memory: cross-sectional and longitudinal explorations in children. Neuropsychology 17, 439–450. 10.1037/0894-4105.17.3.439 12959510

[B11] Hope J. (2006, September 20). Music lessons are an IQ booster for young minds. Mail Online. Available online at: http://www.dailymail.co.uk/news/article-406023/Music-lessons-IQ-booster-young-minds.html (Accessed 3 July, 2015).

[B12] Huffington Post (2011, July 20). Musicians are probably smarter than the rest of us. Huffington Post. Available online at: http://www.huffingtonpost.com/2011/07/20/music-intelligence_n_904124.html (Accessed 3 July, 2015).

[B13] Johnson C. Y. (2013, December 11). Music doesn't make you smarter, Harvard study finds. Boston Globe. Available online at: http://www.bostonglobe.com/lifestyle/health-wellness/2013/12/11/music-makes-you-smarter-right-actually-doesn-harvard-study-finds/DfHtQ1RahjMpFF7C6YolWN/story.html (Accessed 3 July, 2015).

[B14] Klass P. (2012, September 11). Early music lessons have longtime benefits. The New York Times, D6.

[B15a] Mehr S. A. (2013, December 22). Music and Success. The New York Times, SR12.

[B15] Mehr S. A. (2014). Music in the home: new evidence for an intergenerational link. J. Res. Music Educ. 62, 78–88. 10.1177/0022429413520008

[B16] Mehr S. A. Schachner A. Katz R. C. Spelke E. S. (2013). Two randomized trials provide no consistent evidence for nonmusical cognitive benefits of brief preschool music enrichment. PLoS ONE 8:e82007. 10.1371/journal.pone.0082007 24349171 PMC3859544

[B17] Mooallem J. (2010, May 1). The love that daren't squawk its name: when animals come out of the closet. The Independent. Available online from http://www.independent.co.uk/news/science/the-love-that-darent-squawk-its-name-when-animals-come-out-of-the-closet-1957085.html (Accessed 3 July, 2015).

[B18] Neuman S. (2013, December 12). So much for the ‘Mozart effect.’ National Public Radio. Available online at: http://www.npr.org/blogs/thetwo-way/2013/12/12/250542727/so-much-for-the-mozart-effect (Accessed 3 July, 2015).

[B19] O'Neil J. (2003, July 29). More music yields more words. The New York Times, 6.

[B20] Radford T. (2003, September 12). Music improves brain power – in some performers. The Guardian, 2.

[B21] Rauscher F. H. Shaw G. L. Ky K. N. (1993). Music and spatial task performance. Nature 365, 611. 10.1038/365611a0 8413624

[B22] Pew Research Center (2013). The State of the News Media 2013. Available online at: http://www.stateofthemedia.org (Accessed 3 July, 2015).

[B23] Sifferlin A. (2013, December 11). Do, re, mi, fa-get the piano lessons: music may not make you smarter. Time Magazine. Available online at: http://healthland.time.com/2013/12/11/do-re-mi-fa-get-the-piano-lessons-music-may-not-make-you-smarter/ (Accessed 3 July, 2015).

[B24] Sluming V. Barrick T. Howard M. Cezayirli E. Mayes A. Roberts N. (2002). Voxel-based morphometry reveals increased gray matter density in Broca's area in male symphony orchestra musicians. Neuroimage 17, 1613–1622. 10.1006/nimg.2002.1288 12414299

[B25] Thompson W. F. Schellenberg E. G. Husain G. (2001). Arousal, mood, and the Mozart effect. Psychol. Sci. 12, 248–251. 10.1111/1467-9280.00345 11437309

[B26] Trescott J. (1997, August 5). ‘Arts education’ colors debate; foes, proponents of NEA use strategy for different ends. The Washington Post, B01.

[B27] Young L. C. Zaun B. J. VanderWerf E. A. (2008). Successful same-sex pairing in Laysan albatross. Biol. Lett. 4, 323–325. 10.1098/rsbl.2008.0191 18505710 PMC2610150

